# Thrombocytopenia in patients with *Plasmodium vivax* in Colombia is associated with anti-phosphatidylserine autoantibodies and IL-6, IFNγ, IL-10 and TGF-β

**DOI:** 10.1371/journal.pntd.0013284

**Published:** 2025-07-21

**Authors:** María Camila Velasco-Pareja, Catalina Tovar-Acero, Miriam E. Cantero Guevara, Ana Rodriguez, Juan Rivera-Correa, María Fernanda Yasnot-Acosta

**Affiliations:** 1 Bacteriology Department, Universidad de Córdoba, Colombia, Laboratorio de Salud Pública, Grupo de Investigaciones Microbiológicas y Biomédicas de Córdoba, Montería, Córdoba - Colombia; Universidad de Cartagena, Doctorado en Medicina Tropical, Cartagena, Bolivar, Colombia; 2 Medicine Department, Universidad del Sinú, Montería Córdoba, Grupo de Enfermedades Tropicales y Resistencia Bacteriana, Montería, Córdoba, Colombia; 3 Chemistry Department, Universidad de Córdoba, Colombia. Laboratorio de Salud Pública, Grupo de Investigaciones Microbiológicas y Biomédicas de Córdoba, Montería, Córdoba, Colombia; 4 School of Medicine, New York University, New York, New York, United States of America; 5 Biological Sciences Department, New York City College of Technology, City University of New York, Brooklyn, New York, New York, United States of America; 6 Biology PhD Program, City University of New York Graduate Center, New York, New York, United States of America; 7 Bacteriology Department, Universidad de Córdoba, Colombia, Laboratorio de Salud Pública, Grupo de Investigaciones Microbiológicas y Biomédicas de Córdoba, Montería, Córdoba, Colombia; George Washington University School of Medicine and Health Sciences, UNITED STATES OF AMERICA

## Abstract

*Plasmodium vivax* is the most widely distributed human protozoan in the world. It is a causative agent of malaria, with thrombocytopenia being a frequent complication. This study aimed to evaluate the effect of *P. vivax* infection on plasma cytokine/chemokine levels and anti-phosphatidylserine (anti-PS) autoantibodies, to explore their potential role in thrombocytopenia during *P. vivax* malaria in Córdoba, Colombia. We included patients with *P. vivax* malaria and thrombocytopenia (MT); patients with malaria without thrombocytopenia (M) and healthy controls (HC). Plasma cytokines/chemokines (IL-2, IL-4, IL-1β, TNF-α, IL-17A, IL-6, IL-10, IFNγ, IL-12p70, TGF-β1/IP-10, MCP-1, IL-8) were quantified. Evaluation of autoantibodies was performed by ELISA, using phosphatidylserine (PS) as the antigen. IFNγ, IL-6, and IL-10 were found to increase in the MT group (P < 0.05), whereas TGF-β1 was increased in the M group (P < 0.0001). Anti-PS antibody levels were also higher in the MT group and showed a negative correlation with platelet counts. These findings suggest that thrombocytopenia in *P. vivax* malaria may result from autoantibodies targeting phosphatidylserine on activated platelets, driven by a pro-inflammatory cytokine imbalance, with TGF-β1 potentially exerting a protective effect.

## Author summary

Malaria remains a major global health concern, with *Plasmodium vivax* being the most prevalent species in the Americas. Although historically regarded as less severe than *P. falciparum*, *P. vivax* is increasingly associated with severe and complicated malaria, including hematological abnormalities such as thrombocytopenia. Despite its high frequency, the mechanisms underlying *P. vivax*-associated thrombocytopenia are not fully understood. In this study, we found a previously unreported negative correlation between platelet count and autoimmune anti-phosphatidylserine (anti-PS) IgG antibodies, suggesting a role for autoimmunity in disease pathogenesis. This was further supported by a dysregulated cytokine profile, characterized by elevated levels of IFN-γ, IL-6, and IL-10 in patients with thrombocytopenia compared to those without. Notably, higher levels of TGF-β1 were observed in patients without hematological complications, indicating a potential protective and immunomodulatory role. Our findings contribute to a better understanding of the immunopathogenesis of *P. vivax* malaria and may inform future strategies for disease management and prognosis.

## Introduction

Malaria is a disease caused in humans by five species of protozoan parasites of the genus *Plasmodium* [1,2], however, the two most prevalent species in the world are *P. falciparum* and *P. vivax*. According to the World Health Organization, in the Americas, 77% of malaria cases occur in Venezuela, Colombia and Brazil [3]. Studies have shown that *P. vivax* infection, previously considered benign, can cause complicated malaria, a challenge in endemic areas [2,4,5].

Malaria-related thrombocytopenia is generally observed in 24% to 94% of patients with acute malaria and is the most common hematological abnormality in patients with acute malaria [6,7]. Severe thrombocytopenia has been described as the most prevalent severity criterion of *P. vivax* malaria [8,9]. In Colombia, according to the National Institute of Health (INS), hematological complications are the most common [10]; therefore, clinically low platelet counts are often associated with a poor prognosis and prolonged hospital stays [11]. Platelets can contribute to the host’s immune response through various mechanisms such as the secretion of immunomodulatory molecules and cell-cell interactions. Due to this function, they may have protective functions in the host, which in malaria are related to the destruction of the parasite *per se* [12].

Several mechanisms have been reported for malaria-associated thrombocytopenia: oxidative stress damage, phagocytosis, alterations in bone marrow production, cytokine imbalance, platelet activation and aggregation capacity [13,14]. A mechanism that has been proposed for other hematological alterations (anemia) in malaria is autoimmunity mediated by atypical memory B cells (atMBc), which are capable of producing autoantibodies against phosphatidylserine (PS), a molecule exposed in the erythrocyte membrane during malaria, which correlates with anemia [15,16]. Exposure of PS also occurs in activated platelets, which in malaria has been associated with the Ca^2^+-dependent lipid scramblase, which can rapidly move phospholipids back and forth between the two membrane leaflets (flip-flop) [17]. We hypothesize that thrombocytopenia and anemia have similar mechanisms in malaria, due to autoantibodies recognizing PS on platelets and favored by a highly inflammatory environment.

A characteristic feature of *P. vivax* infection is the intense cytokine-mediated proinflammatory stimulation of the immune response observed during the acute phase of the disease [18], characterized by the induction of elevated levels of both pro- and anti-inflammatory cytokines [18,19]. Pro-inflammatory cytokines can favor the clearance of parasitemia by promoting the function of effector cells. Anti-inflammatory cytokines, such as transforming growth factor-beta (TGF-*β*) and IL-10, maintain the balance between the pro-inflammatory and anti-inflammatory responses. If this balance is disrupted, the exaggerated proinflammatory response leads to significant adverse effects associated with severe forms of malaria and a high mortality rate [20]. These may directly affect platelets and other mechanisms that lead to severe malaria, which are not fully understood. We hypothesize that an imbalance of pro-inflammatory and anti-inflammatory cytokines could have a role in promoting thrombocytopenia in *P. vivax* patients.

Platelets have an important role in *P. vivax* infections and their subsequent decrease is a key point in the pathophysiology and clinical course of the disease. The main goal of this study was to evaluate the effects of *P. vivax* infection on cytokine immune response, autoantibodies and their correlation with platelet counts.

## Materials and methods

### Ethics

The enrollment and participation of the subjects were voluntary. Written informed consent was obtained from the participants prior to inclusion in the study. For children and/or disabled persons, the consent was signed by their legal custodian. The project procedures were carried out according to the Resolution No. 008430 from October 4^th^, 1993, Republic of Colombia, Ministry of Health and the Helsinki Declaration and its amendments, the World Medical Association (WMA, Edinburgh, Scotland, October 2000). The Human Ethics Committee from the Health Sciences Faculty of Universidad de Córdoba approved the study development, Act 001, 2016.

### Study site

*Plasmodium vivax* accounts for approximately 72% of malaria cases across Latin America. Brazil, Venezuela, and Colombia together represent 77% of all reported cases in the region [21,22]. Although the prevalence of *Plasmodium falciparum* and *Plasmodium vivax* in Colombia is approximately equal (around 50% each), *P. vivax* is markedly more prevalent in northwest of the country. This epidemiological profile has led our research group to focus primarily on *P. vivax.* Notably, severe malaria cases in Colombia are most frequently associated with *P. vivax* infections, which often present with hematological complications [22,23].

**Fig 1:**
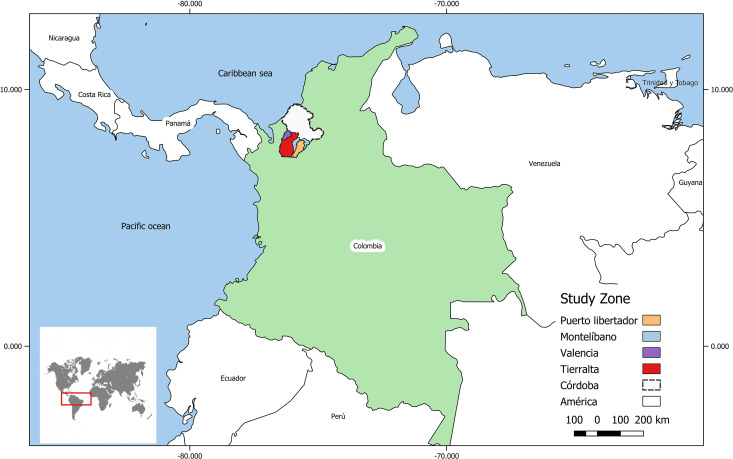
Study Zone. Modified from Rivera-Correa J., *et al, 2020* [25]. Base layer .https://www.colombiaenmapas.gov.co/?e=-77.41829537648094,7.0910832384748215,-74.86397408741858,8.432710278170422,4686&b=igac&l=199&u=23&t=29&servicio=199 under CC BY 4.0 license. The data were processed and visualized using QGIS, an open-source software.

This study was conducted in Córdoba (northwest of Colombia), one of the 32 administrative departments of Colombia, located in the northern Caribbean region of the country. Córdoba accounts for approximately 9% to 15% of all malaria cases reported in Colombia. Over the past seven years, *P. vivax* has accounted for approximately 83% (±4%) of malaria cases in Córdoba [24]. The study participants were residents of the municipality of Tierralta (Fig 1), situated in the southwestern part of the department. Tierralta is located at 8°10’4“N latitude and 76°03’46” W longitude, with an altitude of 51 meters above sea level. It covers a territorial area of 4,728 km², making it the largest municipality in Córdoba, representing 20.3% of the department’s total area. It has a tropical forest and a large dam [21]. Tierralta experiences stable malaria transmission throughout the year and is considered a high-risk area for *Plasmodium* spp., with an annual parasite index (API) of 72.6 cases per 1,000 inhabitants [22,23] and has reported up to 10% of the total malaria cases in the country and represents around 50% of the total malaria cases in Córdoba.

### Study design

We conducted an analytical cross-sectional study using non-probabilistic convenience sampling between October 2017 and March 2019. Patients were recruited from sentinel microscopy sites. Only those diagnosed with *P. vivax* malaria were included. A study group of healthy subjects, inhabitants of the endemic area, afebrile at the time of sampling, without malaria events in the previous six months were included (to avoid bias from latent stages of previous infections, we included only patients who had completed the full *P. vivax* malaria treatment with primaquine in case of prior *P. vivax* infection.). All patients and healthy controls were confirmed by malaria-nested PCR [26]. Children younger than 2 years old, pregnant women, and patients with other clinical conditions were excluded: Leptospirosis (tested by microagglutination with serogroups of *Leptospira interrogans* sensu lato), Dengue (ELISA, Vircell, IgM -ref M1018- and IgG -G1018-, Brucellosis (tested using the Rose Bengal test, Institute Pourquier, Montpellier, France), and febrile antigens (BioSystems, code 33001) were screened in all patient sera. Additionally, malaria caused by *P. falciparum* or mixed *Plasmodium* infections was excluded using nested-PCR according to the Snounou *et al*. protocol [26]. Other exclusion criteria, assessed through medical interview, included urinary tract infections, respiratory diseases, cancer, diabetes, hematological disorders, and autoimmune diseases.

Venipuncture was performed using a vacuum system to obtain 5 - 10 mL of anticoagulated blood during the febrile period and prior to the initiation of antimalarial therapy. A complete automated blood cell count was performed from the whole blood sample.

We included 142 subjects with malaria and 60 healthy controls. Categorized into three groups:

- MT (n= 127): a group of patients with *P. vivax* malaria and thrombocytopenia (defined as platelet count<150,000/µL).
- M (n= 15): group with malaria without any hematological alteration.
- HC (n= 60): individuals living in an endemic area without active malaria episodes or thrombocytopenia.

### Quantification of cytokines and chemokines in plasma

We performed a multiplex bead-based immunoassay for the cytokines IL-2, IL-4, IL-1β, TNF-α, IL-17A, IL-6, IL-10, IFNγ, IL-12p70, TGF-β1and the chemokines IP-10, MCP-1 and IL-8. It was carried out by flow cytometry, using the LEGENDplex kit (Cat No. 740808) BioLegend. The procedure recommended by the manufacturer was followed.

The reading for each sample was determined by interpolation of the concentration found for each analyte on the curve generated by the seven standards for each of the molecules to be quantified between the ranges of 0 - 10,000 pg/ml. The minimum detectable concentration for the 13 molecules to be identified was found to be in the range of 0.3 - 3.06 pg/ml. Samples were run in duplicate on a FACSCalibur (Becton Dickinson, Franklin Lakes, NJ).

### Determination of antibodies by Enzyme-linked Immunosorbent Assay (ELISA).

Previously established protocols were used [25]. Phosphatidylserine (PS) (Sigma) and merozoite surface protein-1 (MSP1) (BEI Resources (National Institute of Allergy and Infectious Diseases NIAID) were used as antigens. Calculations were performed by dividing the optical density (OD) of each sample by the OD of positive control to obtain the relative units (RU) [25].

### Statistical analysis

GraphPad Prism software version 7.00 was used to calculate central tendency measures. Shapiro-Wilk and Kolmogorov-Smirnov tests were used to confirm the distribution of numerical data. The Kruskal-Wallis ANOVA was used to assess statistical significance between groups and the Mann-Whitney test was used to compare two groups; correlations were performed using the Spearman test. A *P*<0.05 was considered significant and 95% confidence intervals were used.

## Results

### Characterization of the patients included in the study.

Male sex predominated with 56%. Adolescents aged 11–15 years were the most affected age group (34.2%), followed by the group aged 16–20 years (20.5%). The occurrence of malaria in older adults was low (6.2%). Mean parasitemia was 5,107p/µL. Regarding hematological parameters, the median platelet count was 98,000/µL (Table 1). No other hematologic alterations were found.

**Table 1:** Characterization of the patients included in the study.

		*MT n = 127	M n = 15	HC n = 60
**Sex**	**Female**	44%	40%	50%
	**Male**	56%	60%	50%
**Age (years) M(IQR)**		15 (11–20)	16 (13–33)	17 (13–34)
**Platelets (x103/µL)** **M(IQR)**		79 (50 – 107)	186 (168 - 273)	282 (248–331)
**Parasite/µL M(IQR)**		2720 (1735 – 5206)	1.400 (1070 – 2000)	–
**Previous malaria episodes M(Max-Min)**		1 (4–0)	1 (4–0)	–

(M = median; IQR = interquartile range; Max = maximum, Min = minimum) MT = malaria+thrombocytopenia; M = malaria; HC = healthy control. * 13% of patients required hospitalization.

**Fig 2:**
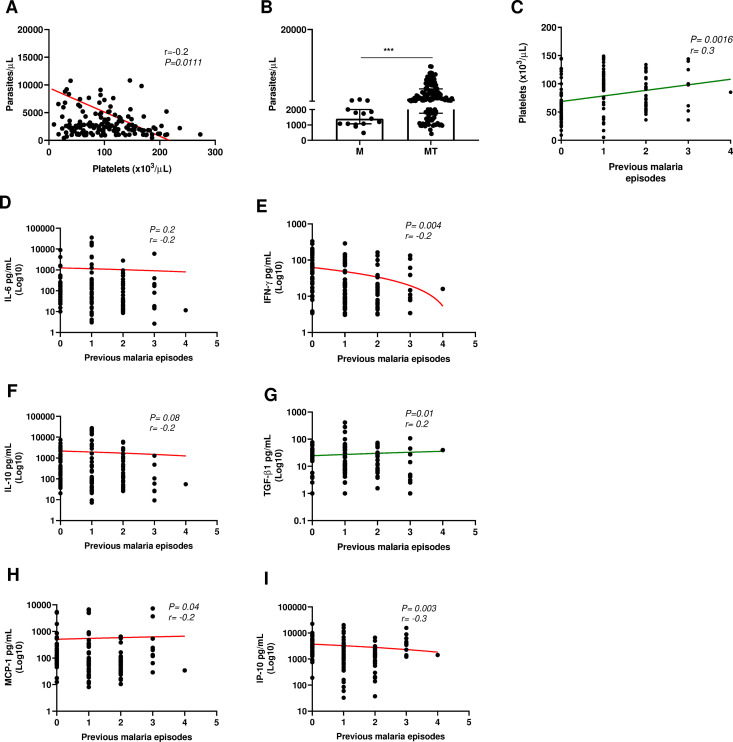
Patient clinical aspects and correlations. A. Spearman correlation between parasites and platelets in malaria patients B. Parasitemia comparison in thrombocytopenic patients and non-thrombocytopenic malaria patients (Mann Whitney analysis). C. Spearman correlation between previous malaria episodes and platelet count D. Spearman correlation between previous malaria episodes and D. IL-6; E.IFN-γ; F. IL-10; G. TGF-β; H. MCP-1; I. IP-10. *** P ≤ 0.0001.

In the MT group, 42% had moderate thrombocytopenia, followed by mild thrombocytopenia at 34% and severe thrombocytopenia at 24%. A positive correlation (r = 0.2, p = 0.001) was observed between previous malaria episodes and platelets. Similarly, IP-10, MCP-1, IL-6 and IFNγ were negatively correlated (P < 0.05) while TGF-β1 was positively correlated (r = 0.2; P < 0.05) with previous malaria episodes. MT group has significantly increased median parasite versus M group and platelets have a negative statistical relation with parasite counts (Fig 2). No significant differences were found between the groups for the other variables shown in Table 1.

### Comparison of cytokines and chemokines in malaria patients

The cytokines that showed significance when comparing the MT and M groups were IL-6, IFNγ, TGF-β1, and IL-10. Increased levels of MCP-1 and IP-10 were observed in malaria patients (S1 Fig). The median plasma concentrations of the cytokines evaluated in this study are presented below (Table 2).

**Table 2:** Median plasma cytokine concentration in the study groups and comparison between groups.

	Plasma concentrations (pg/mL) Median (M) and interquartile ranges (IQR)			ANOVA: Kruskal-Wallis MT + M + HC	Mann Whitney test MT vs M
	MT (n = 127)	M (n = 15)	HC (n = 60)		
**Chemokines**					
**MCP-1**	87 (47–265)	60 (25–193)	47 (20–103)	**P = 0.0007**	NS
**IP-10**	2115 (1198–3940)	1774 (890–3896)	461 (151–892)	**P < 0.0001**	NS
**IL-8**	8.9 (4.6 – 24.3)	6.2 (4–10)	7.3 (4.3–20)	P = 0.45	NS
**Pro-inflammatory cytokines**					
**IL-6**	86.4 (23.1 – 261.2)	19 (9.2–89)	5 (1.7–34)	**P < 0.0001**	**P = 0.006**
**IFN-**γ****	22 (8 – 63.5)	7.1 (4.4–52)	4.5 (4.4–22)	**P < 0.0001**	**P = 0.01**
**IL-12p70**	2.84 (2.4 – 3.9)	2.8 (2.6 – 3.1)	2.8 (2.6 – 3.6)	P = 0.85	NS
**IL-2**	3.6 (2 – 4.1)	2.2 (1.4 – 4.1)	3.6 (1.4 – 4.1)	P = 0.26	NS
**IL-1β**	8.1 (5.2–18)	8 (5.4–34)	7.3 (4–20)	P = 0.7	NS
**TNF-α**	4.4 (4 – 7.4)	4 (3.9 – 4.7)	4 (3.9 – 4.9)	P = 0.27	NS
**IL-17A**	3.5 (2.3 – 7.4)	3.9 (3.4 – 7.9)	3.9 (3.5–10)	P = 0.33	NS
**Anti-inflammatory cytokines**					
**TGF-β1**	11 (4–40)	41 (26–93)	14 (4.2–45)	**P = 0.0012**	**P = 0.0002**
**IL-10**	2115 (1198–3940)	39 (20–99)	3.8 (2.8–18)	**P < 0.0001**	**P = 0.01**
**IL-4**	5.4 (4.2–13)	4.2 (4.1 – 7.1)	4.2 (4.2 – 7.8)	P = 0.03	NS

NS = No significant

**Fig 3:**
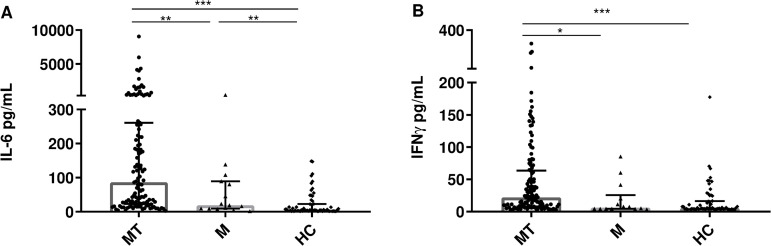
Plasma concentration of cytokines. A. IL-6; B. IFNγ, of the three study groups: malaria and thrombocytopenia (MT) vs malaria (M) vs healthy control (HC). Comparisons were performed using the Kruskal-Wallis test for the overall comparison of the groups and the Mann-Whitney test for the comparison of each group (MT vs M vs HC); *P < 0.05, **P ≤ 0.01, ***P ≤ 0.0001.

IL-6 and IFNγ (Fig 3A and 3B) were higher in patients in the MT group versus the M group.

**Fig 4:**
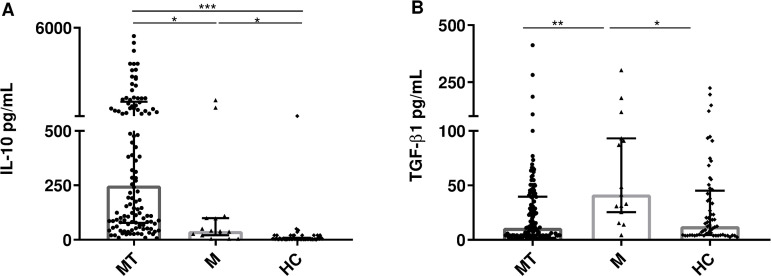
Plasma concentrations of cytokines. A. IL-10; B. TGF-β1, of the three study groups. Comparisons were performed using the Kruskal-Wallis test for the overall comparison of the groups and the Mann-Whitney test for the comparison of each group (MT vs M vs HC); *P ≤ 0.05, **P ≤ 0.01, ***P ≤ 0.0001.

Regarding anti-inflammatory cytokines, IL-10 was found to be elevated in patients with thrombocytopenia compared to those with normal platelet counts (Fig 4A). In the case of TGF-β1 plasma levels were higher in the M versus MT group (Fig 4B).

**Fig 5:**
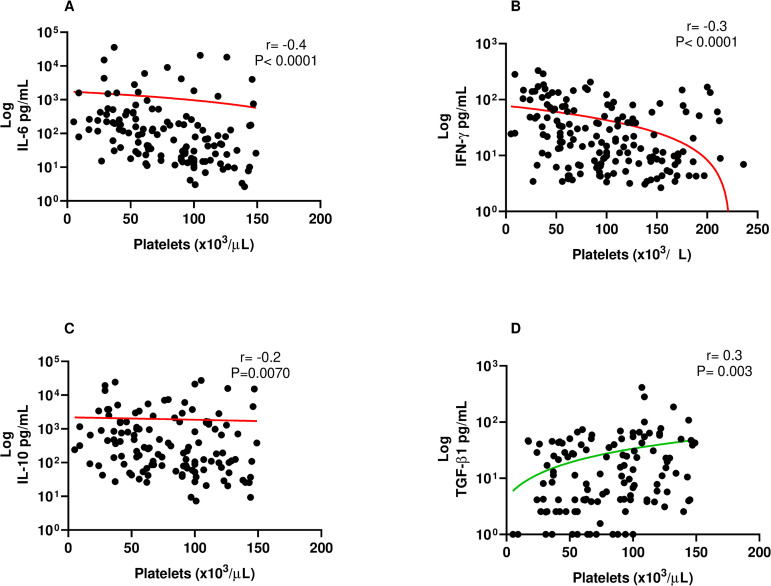
Spearman correlation between platelets count and A. IL-6; B. IFNγ; C. IL-10 and D. TGF-β1. The red line represents a negative correlation; the green line represents a positive correlation.

When we compared the molecules evaluated and the platelet count, we observed that there is a negative correlation of platelets with IL-6; IFNγ; and IL-10 (Fig 5). TGF-β1 has a positive correlation. The other molecules did not correlate with platelets. A low correlation with other cytokines was found (S2 Fig).

### Autoimmune antibodies against phosphatidylserine, but not anti-parasite antibodies, are associated with thrombocytopenia in *P. vivax* malaria infections.

**Fig 6:**
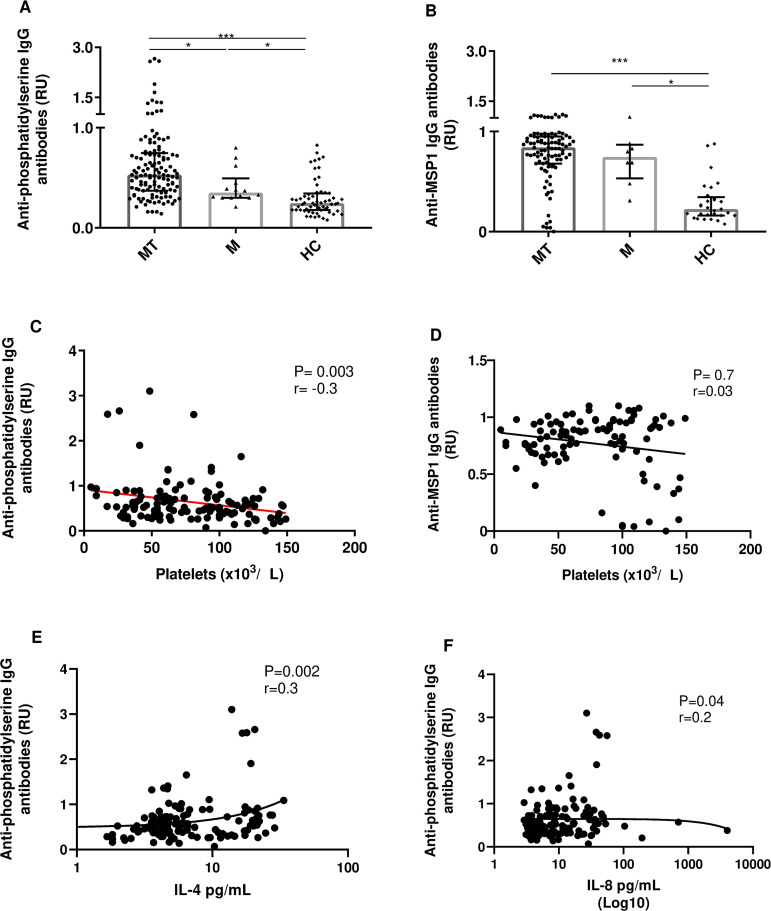
Autoantibodies in patients with malaria and thrombocytopenia. A. Anti-phosphatidylserine IgG antibody concentration, B. Anti-*P. vivax* merozoite surface protein (MSP1) 1 IgG antibody concentration C. Correlation of plasma levels of autoantibodies versus platelet counts in the MT group D. Correlation of plasma anti-MSP1 antibody levels versus platelet counts in the MT group. E. Correlation of IL-4 and autoantibodies anti-PS during vivax malaria. F. Correlation of IL-8 and autoantibodies anti-PS during vivax malaria. A Mann-Whitney test for the comparison of each group (MT vs M vs HC). Correlations were performed using Spearman’s test; a value of P < 0.005 was considered significant.

Analysis of the relative units (RU) of IgG-type autoantibodies against PS in the three study groups showed that the MT group had increased levels of anti-PS antibodies compared to the M group (P < 0.05) (Fig 6). A negative correlation between RU of IgG-type anti-PS antibodies and platelet count was observed (*P* = 0.003), which was not evidenced when the correlation with anti-*P. vivax* -MSP-1 IgG (RU) was evaluated (*P* = 0.7). A cytometry assay was performed and we concluded that patients with severe thrombocytopenia had higher PS expression in the platelet membrane compared to patients with mild thrombocytopenia or malaria (S3 Fig). IL-4 and IL-8 had a positive correlation with anti-PS.

## Discussion

Thrombocytopenia is the most common blood disorder in patients with acute malaria. Severe thrombocytopenia is associated with a higher risk of mortality in both children and adults infected with *P. vivax*. [7]. In this study, we aimed to evaluate the effects of *P. vivax* infection on cytokine and chemokine profiles, as well as autoantibodies, and to examine their association with platelet counts.

Our data confirm that in our patients, *P. vivax* infection is frequently associated with thrombocytopenia and reveal a previously unreported negative correlation between platelet counts and anti-phosphatidylserine (anti-PS) IgG autoantibodies. Studies in Colombia have reported the frequency of thrombocytopenia associated with malaria in a range of 8% to 76% [27–30], with a predominance of severe and moderate thrombocytopenia, similar to that found in our study. The decreased risk of thrombocytopenia associated with previous malaria episodes has been reported in *P. falciparum* [31], but not during *P. vivax* infection and the possible causes and implications need further investigation.

One of the most important findings of our study was the negative correlation between platelet counts and autoimmune anti-PS IgG antibodies. Phosphatidylserine serves as a phagocytic and procoagulant signal on activated platelets and plays a key role in their clearance by endothelial cells [32]. Anti-PS antibodies have been previously described in malaria, where they bind to uninfected erythrocytes and contribute to anemia [16]. Autoantibodies targeting platelet surface molecules such as GPIIb/IIIa, GPIV, GPIb/IX, GPV, and GPIa/IIa have also been reported in human *Plasmodium* spp. infections; however, no significant associations between these antibodies and thrombocytopenia have been found [33,34]. In contrast, our data show a significant association between anti-PS antibodies and lower platelet counts in *P. vivax* infections. This observation suggests a potential mechanism of immune-mediated platelet clearance contributing to *P. vivax*-associated thrombocytopenia and may represent a novel aspect of its pathophysiology.

We also observed a distinct cytokine profile in patients with thrombocytopenia (MT group), characterized by elevated IL-6, IFN-γ, and IL-10 levels, along with reduced TGF-β1. While increased IL-6 and IFN-γ levels have been reported in malaria [35–37], we found a negative correlation between these cytokines and platelet counts. This supports the hypothesis that an unbalanced inflammatory response may contribute to thrombocytopenia. Elevated IFN-γ has been associated with enhanced phagocytic activity in malaria [38] and as a key cytokine in the activation of autoimmune atypical B cells during malaria [39], together with IL-6, has been related to the production of autoantibodies [40]. While these associations suggest a potential mechanistic link to thrombocytopenia, further studies are necessary to establish causality and clarify the underlying pathways.

An important finding of this work is the existing difference in anti-inflammatory cytokines between the groups with high levels of TGF-β1 in the M group. In the MT group, the regulation should be mediated by IL-10, although it seems that this effect is not efficient in preventing thrombocytopenia. The immunomodulatory effect of TGF-β1 has been related to a decrease in the appearance of complicated malaria [41,42], although its influence on hematological parameters remains poorly described. It is important to emphasize that TGF-β1 exerts potent anti-inflammatory functions and has been shown in this work to have a marked protective effect [43]. Further studies are needed to understand the mechanism of regulation mediated by this cytokine, its protective effect against thrombocytopenia and its interaction with IL-10.

In conclusion, our study suggests that thrombocytopenia in *P. vivax* infection may be driven by a combination of immune responses, including autoantibody-mediated recognition of phosphatidylserine on activated platelets and a proinflammatory cytokine dominated by IL-6 and IFN-γ. In contrast, TGF-β1 may exert a protective effect, promoting immune regulation and platelet preservation. These findings provide insights into the immunopathogenesis of thrombocytopenia in *P. vivax* malaria and highlight potential biomarkers for identifying patients at risk for this hematological condition.

The main limitations of our work are the social and geographical characteristics of people with malaria in Córdoba, which make it difficult to carry out randomized probabilistic sampling or a longitudinal study to establish cause and effect. We did not evaluate antibody responses against various platelet surface molecules, such as GPIIb/IIIa, GPIV, GPIb/IX, GPV, and GPIa/IIa, which have been reported as autoantibodies in *Plasmodium* infections and may play a role in thrombocytopenia. Second, this study did not assess activated platelets, platelets exhibiting a procoagulant phenotype, or their ligands for endothelial cells—factors known to influence platelet clearance. Future research incorporating these analyses could provide a more comprehensive understanding of the immunological mechanisms driving thrombocytopenia in *P. vivax* malaria.

## Supporting information

S1 Fig (Related to Figs 3 and 4). Plasma concentrations of chemokines IP-10; MCP-1, of the three study groups. Comparisons were performed using the Kruskal-Wallis test for the overall comparison of the groups and the Mann-Whitney test for the comparison of each group (MT vs M vs HC); * P ≤ 0.05, ** P ≤ 0.01, *** P ≤ 0.0001.
(TIF)

S2 Fig (Related to Fig 5). Correlation of plasma A.MCP-1; B. IP-10; C.IL-4 levels versus platelet counts in the MT group. Correlations were performed using Spearman’s test; a value of P < 0.005 was considered significant.
(TIF)

S3 Fig (Related to Fig 6). Phosphatidylserine expression in platelets (Gated on CD41a+). Evaluation of Phosphatidylserine Exposure in Platelets of Malaria Patients and its Relationship with Thrombocytopenia. Additionally, we evaluate the expression of phosphatidylserine in purified platelets from malaria patients and healthy controls. We included one patient with malaria (platelet count ≥ 150,000/µL); one patient with malaria and mild thrombocytopenia (platelet count <150,000/µL); one patient with malaria and severe thrombocytopenia (platelet count <50,000/µL) and a healthy volunteer from the endemic area were included for this evaluation. All samples were treated in the same way. To obtain platelets, whole blood was centrifuged at 1200g for 10 minutes. Platelet-rich plasma was separated and resuspended in 1X PBS. Activated platelets were used as a control, stimulated with 1 unit of human alfa thrombin (Enzyme Research Laboratories Cat. No. HT1002a) per one minute at 37°C prior to staining. Purified platelets were labeled with anti-CD41a-PE (Cat. No. 557297 BD Pharmigen), and Annexin V-FITC (Cat. No. 640905 BioLegend) was used as a PS marker. Labeling was performed according to the manufacturer’s instructions; 100000 events were acquired. All flow cytometry was performed on a FACSLyric (Becton Dickinson) and analyzed using FlowJo version 10. It was found that a patient with malaria and severe thrombocytopenia exposed 1.2 times more PS on the platelet membrane versus those without thrombocytopenia and/or mild thrombocytopenia.
(TIF)

S1 Data This file contains the datasets used to generate the figures and tables presented in the main manuscript. (ZIP)
