# Supplementary figures and images for: Thrombocytopenia in patients with Plasmodium vivax in Colombia is associated with anti-phosphatidylserine autoantibodies and IL-6, IFNγ, IL-10 and TGF-β
Source: PLoS Negl Trop Dis. 2025 Jul 21;19(7):e0013284. doi: 10.1371/journal.pntd.0013284 (PMC12279092; doi:10.1371/journal.pntd.0013284)

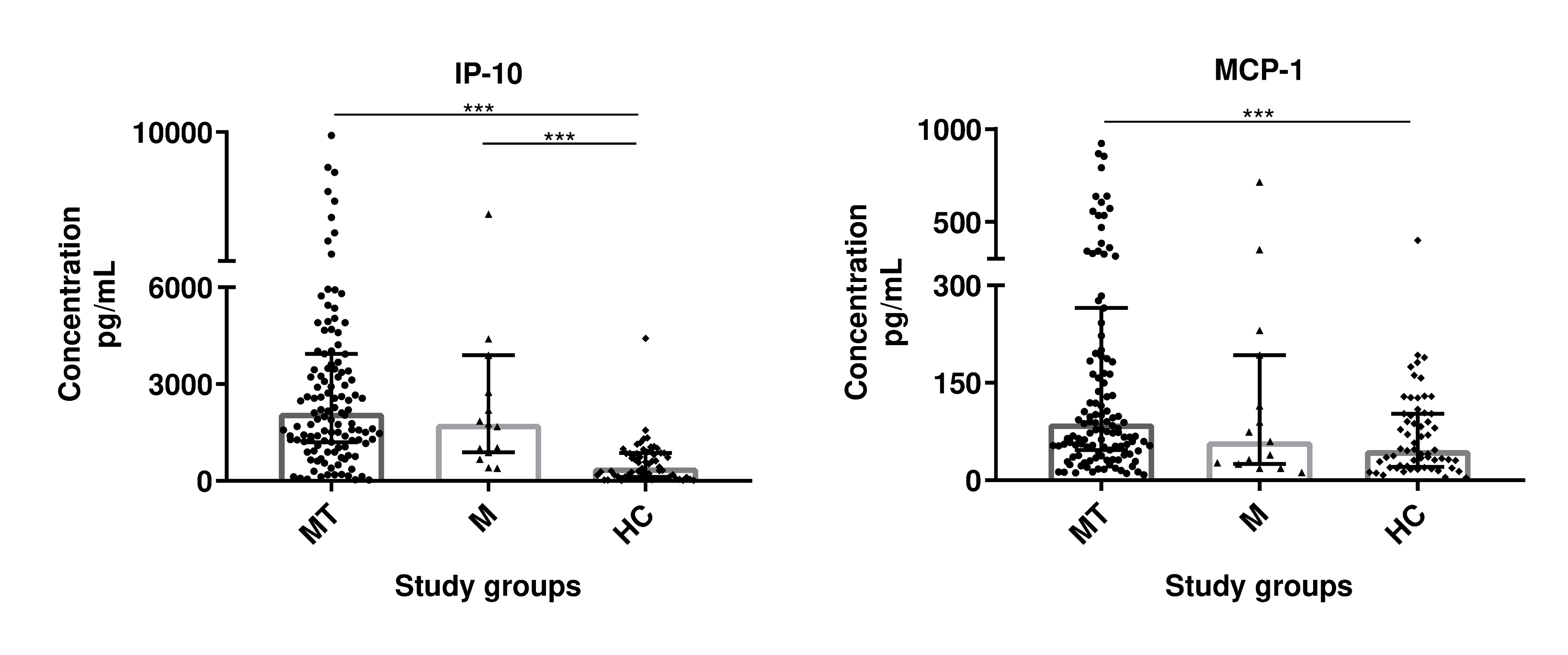

Supplement: S1 Fig — (Related to Figs 3 and 4). Plasma concentrations of chemokines IP-10; MCP-1, of the three study groups. Comparisons were performed using the Kruskal-Wallis test for the overall comparison of the groups and the Mann-Whitney test for the comparison of each group (MT vs M vs HC); * P ≤ 0.05, ** P ≤ 0.01, *** P ≤ 0.0001. (TIF) [file pntd.0013284.s001.tif]

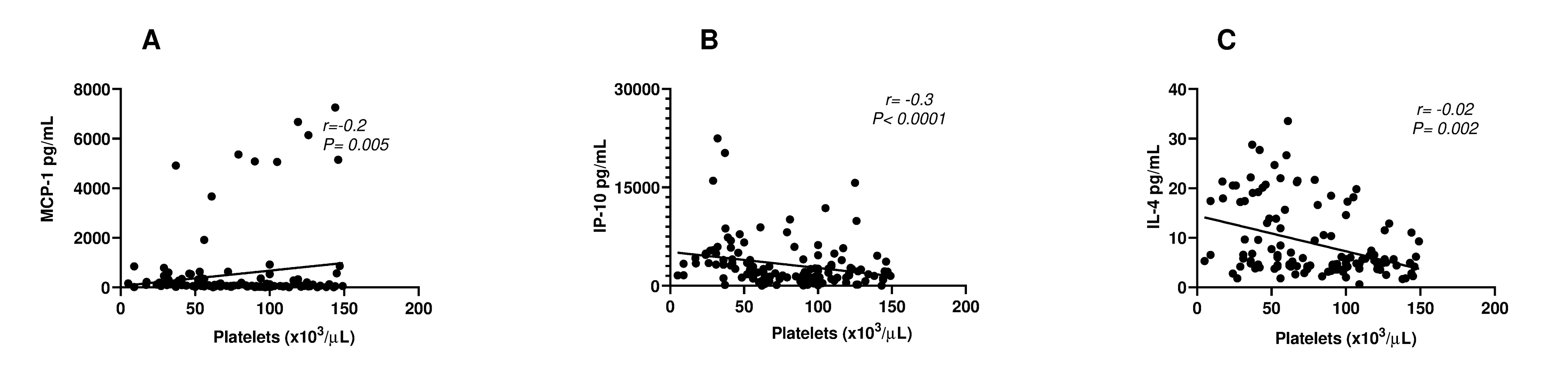

Supplement: S2 Fig — Correlations were performed using Spearman’s test; a value of P < 0.005 was considered significant. (TIF) [file pntd.0013284.s002.tif]

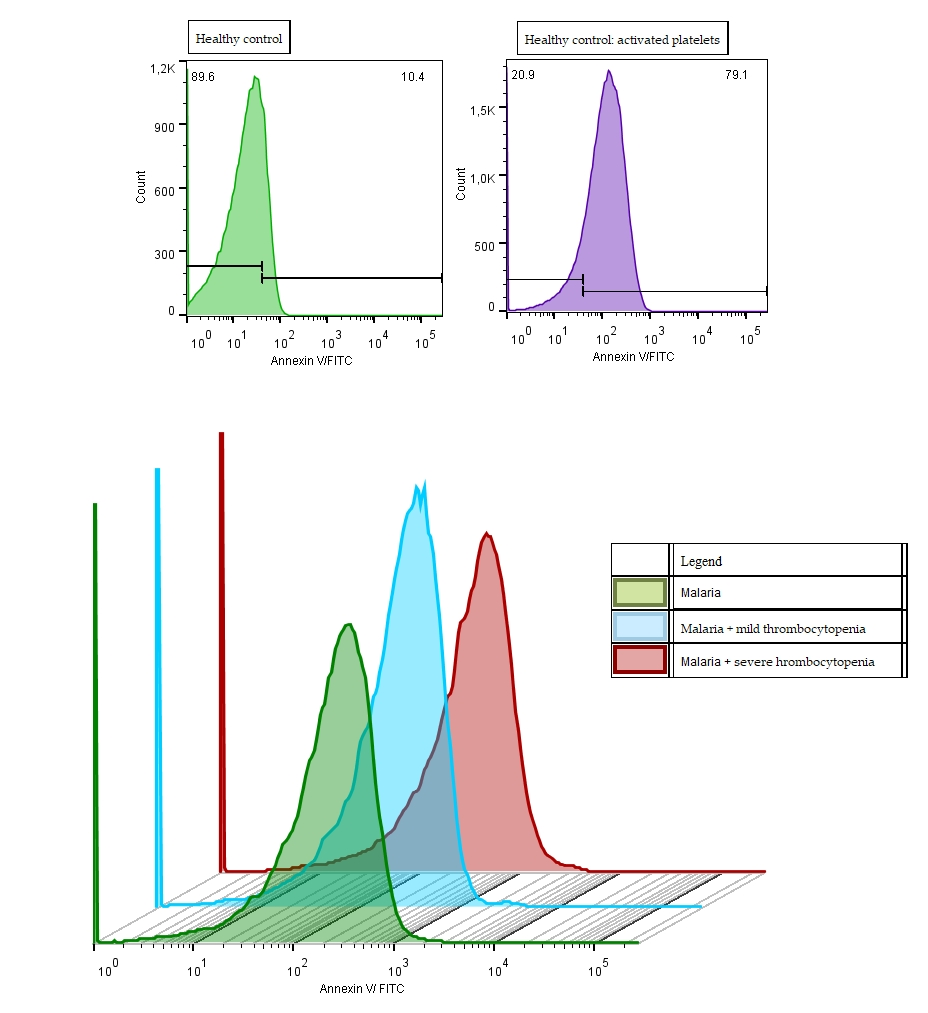

Supplement: S3 Fig — Evaluation of Phosphatidylserine Exposure in Platelets of Malaria Patients and its Relationship with Thrombocytopenia. Additionally, we evaluate the expression of phosphatidylserine in purified platelets from malaria patients and healthy controls. We included one patient with malaria (platelet count ≥ 150,000/µL); one patient with malaria and mild thrombocytopenia (platelet count <150,000/µL); one patient with malaria and severe thrombocytopenia (platelet count <50,000/µL) and a healthy volunteer from the endemic area were included for this evaluation. All samples were treated in the same way. To obtain platelets, whole blood was centrifuged at 1200g for 10 minutes. Platelet-rich plasma was separated and resuspended in 1X PBS. Activated platelets were used as a control, stimulated with 1 unit of human alfa thrombin (Enzyme Research Laboratories Cat. No. HT1002a) per one minute at 37°C prior to staining. Purified platelets were labeled with anti-CD41a-PE (Cat. No. 557297 BD Pharmigen), and Annexin V-FITC (Cat. No. 640905 BioLegend) was used as a PS marker. Labeling was performed according to the manufacturer’s instructions; 100000 events were acquired. All flow cytometry was performed on a FACSLyric (Becton Dickinson) and analyzed using FlowJo version 10. It was found that a patient with malaria and severe thrombocytopenia exposed 1.2 times more PS on the platelet membrane versus those without thrombocytopenia and/or mild thrombocytopenia. (TIF) [file pntd.0013284.s003.tif]
